# Metagenomic and metabolomic analysis of the early gut microbiota development in *Nipponia nippon*

**DOI:** 10.3389/fmicb.2026.1909232

**Published:** 2026-10-08

**Authors:** Hongqing Bai, Guoqiang Qiu, Jian Shi, Yu Xue, Wenwen Dai, Qian Liu, Xiaolong Zhou, Ke He

**Affiliations:** 1 Deqing County Bureau of Natural Resources and Planning, Huzhou, China; 2 Deqing County Ecological Forestry Comprehensive Service Center, Huzhou, China; 3 Zhejiang Province crested ibis Rescue and Conservation Base, Huzhou, China; 4 Key Laboratory of Applied Technology on Green-Eco-Healthy Animal Husbandry of Zhejiang Province, College of Animal Science and Technology, College of Veterinary Medicine, Zhejiang A&F University, Hangzhou, China

**Keywords:** crested ibis, early development, gut microbiota, metabolomics, metagenomics

## Abstract

**Introduction:**

The crested ibis (Nipponia nippon) is an endangered bird whose early gut microbiota development remains poorly understood. To address this gap, this study integrated metagenomics and metabolomics to investigate the dynamic succession of gut microbiota and metabolism in naturally hatched chicks during early development.

**Methods:**

Fecal samples were collected from naturally hatched crested ibis chicks aged 3 to 33 days and divided into groups Z3, Z8, Z13, Z18, Z23, Z28, and Z33. Integrated metagenomics and metabolomics were used to assess microbial gene richness, alpha and beta diversity, taxonomic composition, functional profiles, differentially expressed metabolites, pathway enrichment, and microbe-metabolite correlations.

**Results:**

Gut microbial gene richness and alpha diversity increased from Z3 to Z13, then fluctuated and stabilized, with Z13-Z18 representing a critical colonization window. Beta diversity analysis revealed progressive structural remodeling with age. At the phylum level, Pseudomonadota dominated early stages, while Bacillota increased over time. At the genus level, Plesiomonas, Clostridium, and Escherichia were prevalent; Enterobacter and Ligilactobacillus decreased by Z33, whereas Campylobacter, Cetobacterium and Proteus increased. Functional profiling indicated that Z3 exhibited high metabolic activity, including carbohydrate, amino acid, and energy metabolism, while Z33 showed reduced but more specialized functions. Metabolomics identified numerous differentially expressed metabolites across age groups, with pathway enrichment in cofactor biosynthesis, nucleotide metabolism, and amino acid metabolism. Correlation analysis revealed opposite metabolic associations: Corynebacterium was positively correlated with most metabolites, whereas Enterobacter, Citrobacter, and Klebsiella showed negative correlations.

**Discussion:**

These findings reveal dynamic succession of gut microbiota and metabolism during early development in crested ibises. The identification of a critical colonization window and age-related microbial and metabolic shifts provides fundamental insights for improving captive breeding and conservation of this endangered species.

## 1 Introduction

The crested ibis (*Nipponia nippon*) is a rare East Asian wading bird famous for its elegant white plumage, wispy head crest, and dramatic conservation comeback from the brink of extinction. The crested ibis, also called the Asian crested ibis, is the only member of its genus and is native to eastern Asia. Historically it lived across China, Japan, the Korean Peninsula, and parts of the Russian Far East, but its numbers collapsed in the 20th century due to habitat loss, hunting, and agricultural change. Once thought nearly extinct in the wild, it is now classified as endangered after intensive protection and reintroduction programs (Zhang et al., 2015; Zou et al., 2024; Yu et al., 2015). The Xiazhu Lake Wetland, characterized by a mosaic of islands and abundant aquatic prey (loaches, eels, and frogs), provided an ideal habitat for the crested ibis (Li et al., 2025; Liu et al., 2020). By late 2022, the local population exceeded 600 individuals, with nearly half reintegrated into the wild. Currently, Deqing represents the largest population of crested ibis in China.

Gut microbes in intestinal microorganisms during early life have a strong impact on birds‘ growth, organs and immune system development, and disease resistance. Disturbances in this period can leave long-lasting effects into adulthood (Wu et al., 2024; Sun et al., 2022; Al Hakeem et al., 2023). Bird embryos develop in eggs with very low microbial exposure, and gut colonization begins via microbes from the hen's reproductive tract, the egg surface, and the nest environment even before and immediately after hatching. After hatch, the gut microbiota changes rapidly over days to weeks, with successive waves of bacteria and increasing diversity as chicks begin feeding and interacting with parents and the environment (Huang et al., 2023; Diez-Méndez et al., 2023; Teyssier et al., 2018). Gut microbes support digestion by helping break down complex carbohydrates, producing short-chain fatty acids, and contributing to vitamin supply, which in turn supports rapid weight gain in young birds. In broiler chickens, variation in early gut microbiota composition correlates with average daily gain and feed efficiency, indicating that microbial communities influence growth performance (Teyssier et al., 2018). Microbes also help shape intestinal morphology: they influence villus size, crypt depth, mucous layer production, and goblet cell function, which are essential for nutrient absorption and barrier integrity. Studies in chicks show that early colonizers modulate the development of gut barrier components and mucosal defenses, affecting how well the intestine excludes pathogens and toxins (Broom and Kogut, 2018; Rodrigues et al., 2021). The gut microbiome is crucial for maturation of both innate and adaptive immunity in birds, helping train immune cells to distinguish between harmless and harmful microbes. Experiments using defined microbial consortia in chicks demonstrate that controlled early colonization accelerates immune system maturation and improves resistance to enteric pathogens (Zenner et al., 2021; Al Hakeem et al., 2023).

Early life is a critical window of opportunity. Changes in the microbiota during this period can produce lasting effects on metabolism, immune responsiveness, and disease susceptibility later in life. Both poultry and wild bird studies suggest that the composition and diversity of gut microbiota in the first weeks influence later growth, health, and survival probabilities (Li et al., 2022). This project utilizes naturally hatched crested ibis, and conducts metagenomic and metabolomic studies to investigate the effects of different days after hatching on the intestinal flora and digestive absorption functions of the ibises.

## 2 Materials and methods

### 2.1 Materials and experimental design

Fresh fecal samples were collected from crested ibises' chicks at the Xiazhu Lake Crested Ibis Breeding Center (Huzhou, China). Chicks aged 3 to 33 days were provided with diets of identical composition throughout the study period. This study used a longitudinal cohort design. Crested ibis' chicks were artificially reared and monitored from 3 to 33 days post-hatching. The number of successfully sampled individuals varied across time points (*N* = 4–8) due to failure to collect excreta within the monitoring interval, or insufficient DNA quality. The samples were categorized into seven groups based on developmental stage: Z3 (3 days after hatching, *N* = 8), Z8 (8 days after hatching, *N* = 6), Z13 (13 days after hatching, *N* = 8), Z18 (18 days after hatching, *N* = 4), Z23 (23 days after hatching, *N* = 8), Z28 (28 days after hatching, *N* = 8), Z33 (33 days after hatching, *N* = 7). Chicks were naturally hatched and subsequently hand-reared (artificially fed) with a standardized formula at the breeding center (Supplementary Table S1). To ensure safety and sterility, the chicks were stabilized by experienced handlers while researchers collected swab samples using sterile equipment.

### 2.2 Microbial diversity analysis

Genomic DNA Library Construction and Sequencing. Genomic DNA (1 μg) was stochastically fragmented to an average size of 350 bp using a Covaris ultrasonic disruptor. Sequencing libraries were constructed following standard protocols, including end repair, A-tailing, adapter ligation, purification, and PCR amplification. Library integrity and insert size were verified via Automated Capillary Electrophoresis (AATI) analysis, and the effective concentration (>3 nM) was quantified using qPCR. Qualified libraries were pooled based on target data outputs and sequenced on an Illumina platform using a paired-end 150 bp (PE150) strategy. The raw sequencing reads have been deposited in the NCBI Sequence Read Archive (SRA) database (BioProject Accession Number: PRJNA1406017).

Gene Catalog Construction and Taxonomic Annotation. The CD-HIT software was utilized to eliminate redundancy from the ORF predictions, creating a non-redundant initial gene catalog. To minimize the influence of contaminant sequences, especially in low-biomass samples, contaminant ASVs were identified and removed using the R package decontam (version 1.18.0) with both the “frequency” method (using post-PCR DNA concentration as a predictor) and the “prevalence” method (comparing biological samples to negative controls). ASVs present in negative controls with relative abundance >1% were flagged as potential contaminants and removed if their abundance in samples was strongly correlated with blank abundance. Additionally, ASVs with total counts < 10 across all samples or present in only one sample were excluded to reduce noise. Host-derived reads were identified by alignment to the chicken reference genome (Galgal6) using Bowtie2 and removed prior to taxonomic classification. Clean reads from each sample were re-aligned to this catalog using Bowtie2. Genes with a count of two or fewer reads were filtered out to finalize the unigene catalog. Gene abundance was calculated based on aligned read counts and normalized by gene length. For taxonomic classification, unigenes were aligned against the Micro_NR database (comprising bacteria, fungi, archaea, and viruses) using DIAMOND. The lowest common ancestor (LCA) algorithm was applied to assign taxonomic annotations. Taxonomic abundance and diversity were subsequently analyzed using dimensionality reduction methods (PCA, PCoA), community difference testing (ANOSIM), and biomarker discovery tools (including MetaGenomeSeq, LEfSe, and Random Forest models).

Functional profiling was conducted by aligning unigenes against multiple comprehensive databases—including KEGG, eggNOG, CAZy, VFDB, and PHI—using DIAMOND. The Best Hit (highest score) was selected to calculate relative functional abundances. Statistical analyses, such as PCA, NMDS, and LEfSe, were applied to assess functional divergence between groups. Finally, antimicrobial resistance was analyzed by aligning unigenes to the CARD database using the Resistance Gene Identifier (RGI). Mobile genetic elements (MGEs) were profiled by comparing sequences against the ISfinder, INTEGRALL, and plasmid databases to quantify MGE abundance for heatmap visualization. Functional and Resistance Gene Annotation. Functional profiling was conducted by aligning unigenes against multiple comprehensive databases—including KEGG, eggNOG, CAZy, VFDB, and PHI—using DIAMOND. The best BLAST hit was selected to calculate relative functional abundances. Statistical analyses, such as PCA, NMDS, and LEfSe, were applied to assess functional differences between groups.

### 2.3 Metabolome profiling analysis

Fecal samples of crested ibis (50 mg) were transferred to 2 mL centrifuge tubes equipped with 6 mm diameter grinding beads. Metabolite extraction was performed by adding 400 μL of an extraction solvent (methanol:water = 4:1, v/v) containing 0.02 mg/mL of L-2-chlorophenylalanine as an internal standard. Samples were homogenized using a Wonbio-96c frozen tissue grinder (Shanghai Wanbo Biotechnology Co., Ltd.) for 6 min (at −10 °C, 50 Hz), followed by low-temperature ultrasonic extraction for 30 min (at 5 °C, 40 kHz). Subsequently, the samples were incubated at −20 °C for 30 min and centrifuged for 15 min (at 4 °C, 13,000 × g). The resulting supernatant was transferred to autosampler vials for LC-MS/MS analysis. Raw data preprocessing was conducted using Progenesis QI software (Waters Corporation, Milford, USA). Metabolites were identified by querying the Human Metabolome Database (HMDB; http://www.hmdb.ca/), METLIN (https://metlin.scripps.edu/), and the Novogene Database. All raw data were deposited in the CNCB GSA database (accession number OMIX014467).

### 2.4 Statistical analyses

Metagenomic Bioinformatics Analysis. Raw Illumina sequencing data were preprocessed using fastp to generate clean reads. Specifically, reads containing adapter contamination, excessive ambiguous nucleotides (>10%), or a high proportion of low-quality bases (>50%) were filtered out. To mitigate host DNA interference, clean reads were aligned to the *Nipponia nippon* reference genome using Bowtie2, and all host-derived sequences were discarded. The remaining high-quality reads were assembled into contigs using MEGAHIT. Open reading frames (ORFs) were predicted for sequences ≥500 bp using MetaGeneMark, and predicted ORFs shorter than 100 nt were excluded from subsequent analyses.

Metabolomic Analysis. The processed data matrix was uploaded to the Novogene Cloud Platform (https://magic-plus.novogene.com/#/tool/list) for comprehensive statistical analysis. Initial preprocessing involved retaining metabolic features detected in at least 80% of samples within any experimental group. For samples with metabolite levels below the lower limit of quantification, missing values were imputed using the minimum detected value. To account for variations in sample preparation and instrument instability, peak intensities were normalized using the sum normalization method. Furthermore, variables in Quality Control (QC) samples with a relative standard deviation (RSD) > 30% were excluded. The remaining variables were log10-transformed to stabilize variance for downstream analysis. Multivariate statistical analyses, including Principal Component Analysis (PCA) and Orthogonal Partial Least Squares Discriminant Analysis (OPLS-DA), were performed using the R package “ropls” (Version 1.6.2). Model stability was validated using 7-fold cross-validation. Significantly different metabolites (SDMs) were identified based on a Variable Importance in Projection (VIP) score > 1 from the OPLS-DA model and a *p*-value < 0.05 from Student's *t*-test. These SDMs were mapped to biochemical pathways using the KEGG database (http://www.genome.jp/kegg/). Pathway enrichment analysis was conducted using the Python scipy.stats package to identify the biological pathways most relevant to the experimental treatments. For pathway enrichment analysis, only metabolites that could be reliably mapped to endogenous metabolic pathways in the KEGG database were included. Putatively annotated exogenous compounds (e.g., synthetic drugs, pesticides, or other xenobiotics) were excluded from pathway analysis, as they are not involved in host endogenous metabolic regulation and could introduce artifacts.

## 3 Results

### 3.1 The difference in the number of genes

To investigate the impact of different age stages on the gut microbiota composition of crested ibises, metagenomic sequencing was performed. The samples were divided into seven groups (Z3 = 3 days old; Z8 = 8 days old; Z13 = 13 days old; Z18 = 18 days old; Z23 = 23 days old; Z28 = 28 days old; Z33 = 33 days old). Gene counts were analyzed in each group, revealing differences in gene counts among crested ibises of different ages (Figure 1A). Overall, the number of gut microbiota genes initially increased and then fluctuated with increasing age of the crested ibis. Among them, Z18 had the highest median gene count of 843,877, indicating a high gene richness in the gut microbiota at this stage. Z13 and Z23 also had relatively high gene counts (medians of 618,409 and 531,102, respectively), suggesting that the crested ibis gut microbiota gradually becomes richer and more complex during growth and development (Figure 1A). In contrast, Z3 and Z8 had relatively low gene counts overall (medians of 395,449 and 393,057), which may be related to the fact that the gut microbiota was not fully established in the early stages of hatching. Z28 had the lowest gene count (median of 289,331), suggesting that the gene richness of the gut microbiota was relatively low at this stage. From the perspective of intragroup differences, the groups Z13, Z18, and Z33 were obviously different in the number of genes among samples at these age stages, suggesting that there are some differences in the gut microbiota structure between individuals. The microbiota structure is relatively stable but the overall complexity is low.

**Figure 1:**
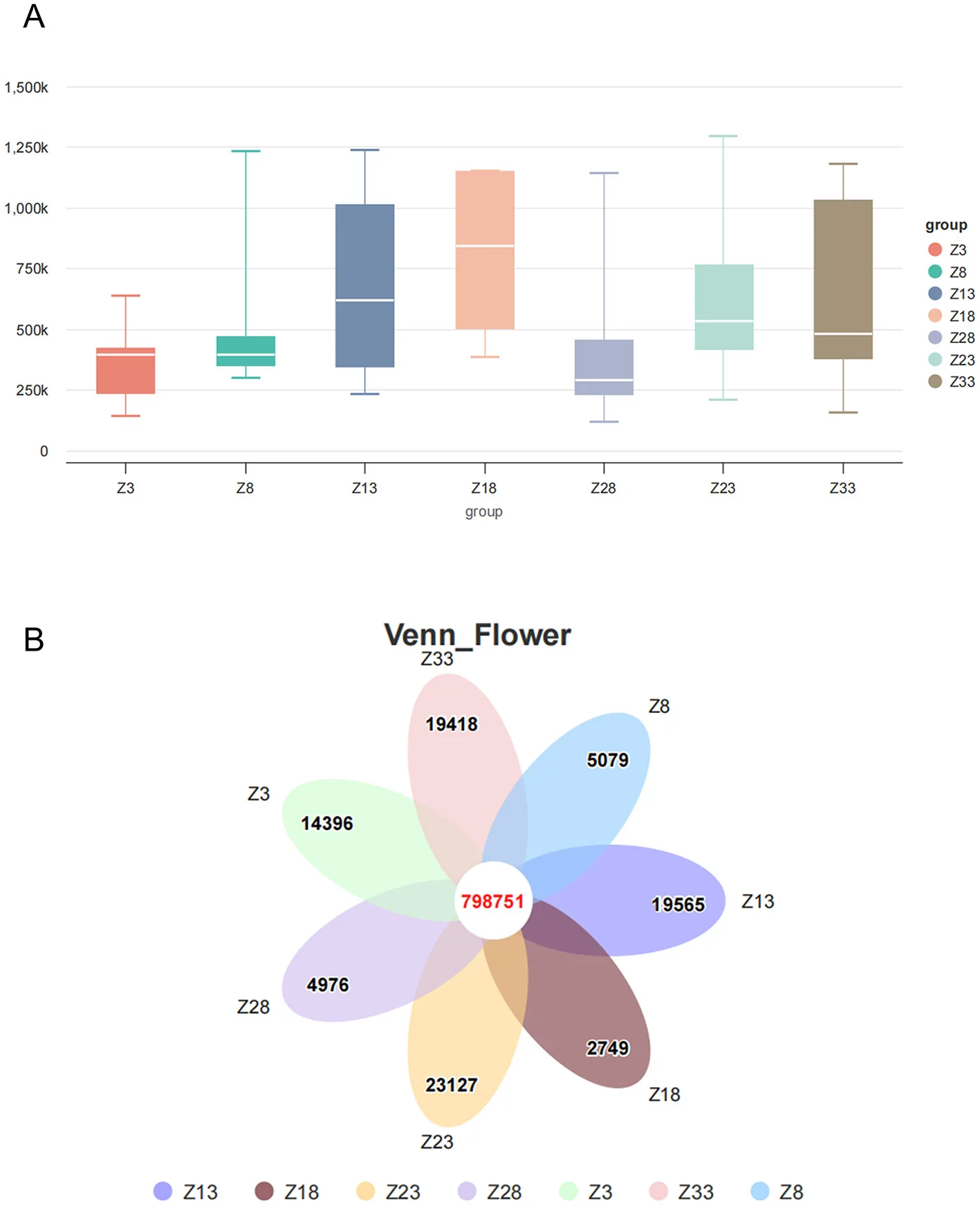
Analysis of gene amount differences in metagenomics. **(A)** The difference of the number of genes in the early gut microbiota development. Z3 (3 days after birth); Z8 (8 days after birth); Z13 (13 days after birth); Z18 (18 days after birth); Z23 (23 days after birth), Z28 (28 days after birth), Z33 (33 days after birth). **(B)** The common and unique genetic information among different groups was plotted using a Venn diagram or a petal diagram.

Overall, the gut microbiota of crested ibises gradually establishes and becomes more complex with increasing age, with the 13–18-day age period likely being a crucial period for rapid microbiota development. The Venn diagram shows the number of common and endemic species across multiple samples. The numbers of endemic genes in groups Z3, Z8, Z13, Z18, Z23, Z28, and Z33 were 14,396, 5,079, 19,565, 2,749, 23,127, 4,976, and 19,418, respectively (Figure 1B).

### 3.2 The diversity of gut microbiota in the crested ibis

Regarding gut microbiota diversity, alpha diversity analysis was performed on the samples (Figures 2A–E). The ACE index, Chao1 index, and Observed_species index reflects the species richness of the gut microbiota. As shown in Figures 2A–C, the richness index of group Z13 is generally higher, indicating a relatively rich number of gut microbiota species at this stage; while the richness of groups Z3 is relatively lower. The Shannon index and Simpson index are used to reflect gut microbiota diversity and evenness (Figures 2D, E). The results show that the Shannon index of group Z33 is higher, indicating that the gut microbiota at 33 days of age has high diversity, while the diversity of group Z23 is relatively lower, which may be related to the increase of dominant bacterial groups. Overall, with increasing age, the gut microbiota diversity of crested ibises shows a trend of gradually increasing and then stabilizing.

**Figure 2:**
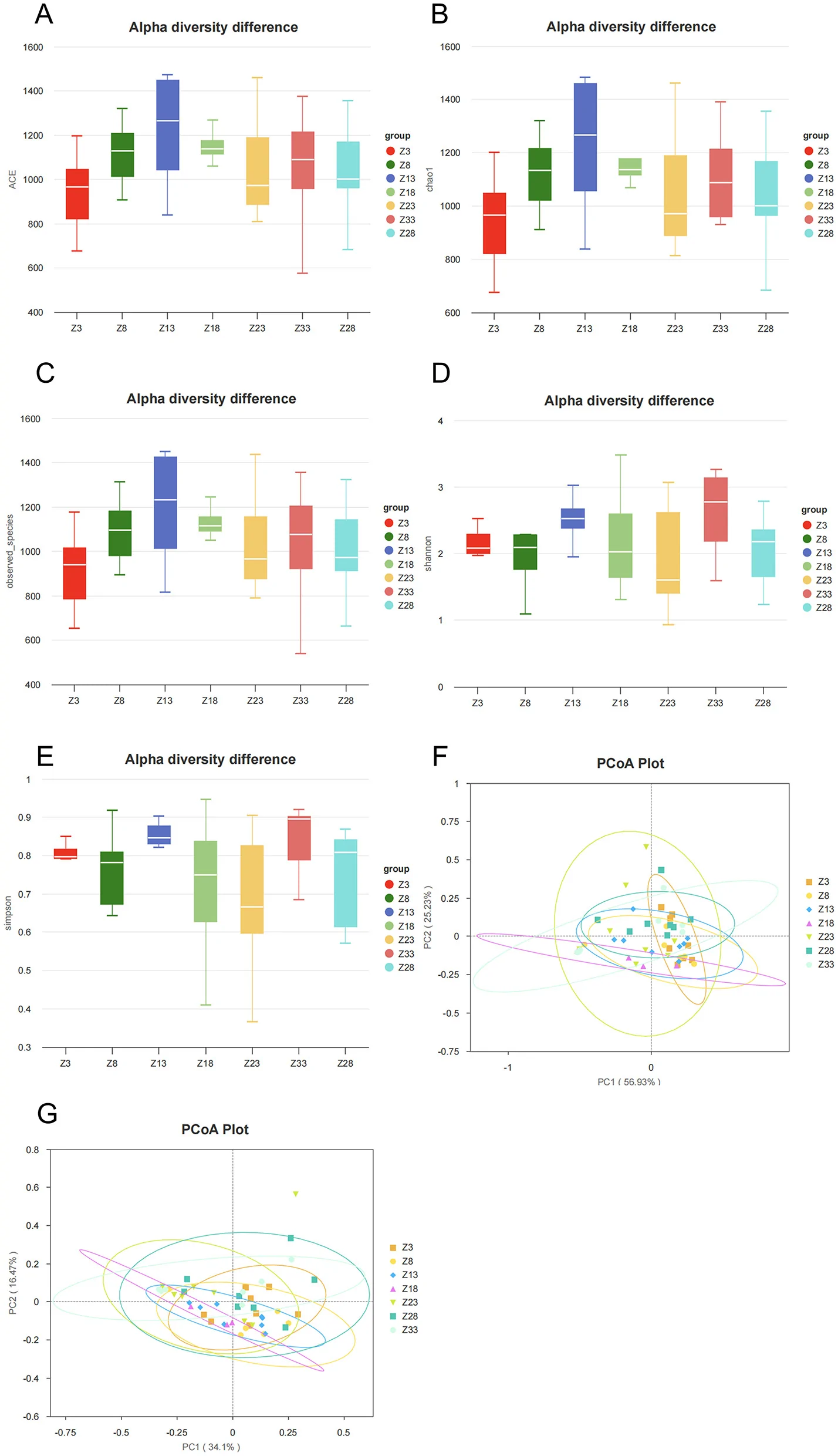
α-diversity and β-diversity of fecal microbiota in crested ibis. **(A)** ACE index. **(B)** CHAO1 index. **(C)** Observed_species index. **(D)** Shannon index. **(E)** Simpson index. **(F)** PCoA analysis at the phylum level. **(G)** PCoA analysis at the genus level.

Furthermore, this study assessed the β-diversity of samples from different age groups using principal coordinate analysis (PCoA). At the phylum level (Figure 2F), the first principal axis (PC1) and the second principal axis (PC2) explained 56.93% and 25.23% of the community differences, respectively. While there was some overlap between samples from different age groups in two-dimensional space, samples within the same group generally showed a clustering trend, indicating a similarity in the gut microbiota structure at different stages, the R-value and significance *P*-value of ANOSIM were shown in Supplementary Table S2. In the genus-level PCoA analysis (Figure 2G), PC1 and PC2 explained 34.1% and 16.47% of the differences, respectively. The sample distribution was relatively more dispersed, indicating that at a finer taxonomic level, the differences in microbiota structure between different age stages were more pronounced, the R-value and significance *P*-value of ANOSIM were shown in Supplementary Table S3.

In summary, as crested ibises age, their gut microbiota gradually evolves from simple to complex, undergoing continuous dynamic adjustments during growth. The period from 13 to 18 days of age exhibits high microbiota richness, potentially representing a crucial period for rapid establishment of the gut microbiota, while the microbiota structure gradually stabilizes in the later stages.

### 3.3 Gut microbial composition and function

At the phylum level, there were many differences in the community composition of the gut microbiota of crested ibises of different ages. The results of the relative abundance histogram at the phylum level showed that the gut microbiota of each sample was mainly composed of phyla such as Pseudomonadota and Bacillota. Pseudomonadota had a higher relative abundance in most samples and was the dominant phylum. The relative abundance of Pseudomonadota was relatively high in Z3, Z8, Z13, and Z18 groups, while the relative abundance of Bacillota showed an increasing trend with increasing age, indicating that the gut microbiota structure gradually changed and tended to stabilize with increasing age (Figures 3A, B, Supplementary Table S4).

**Figure 3:**
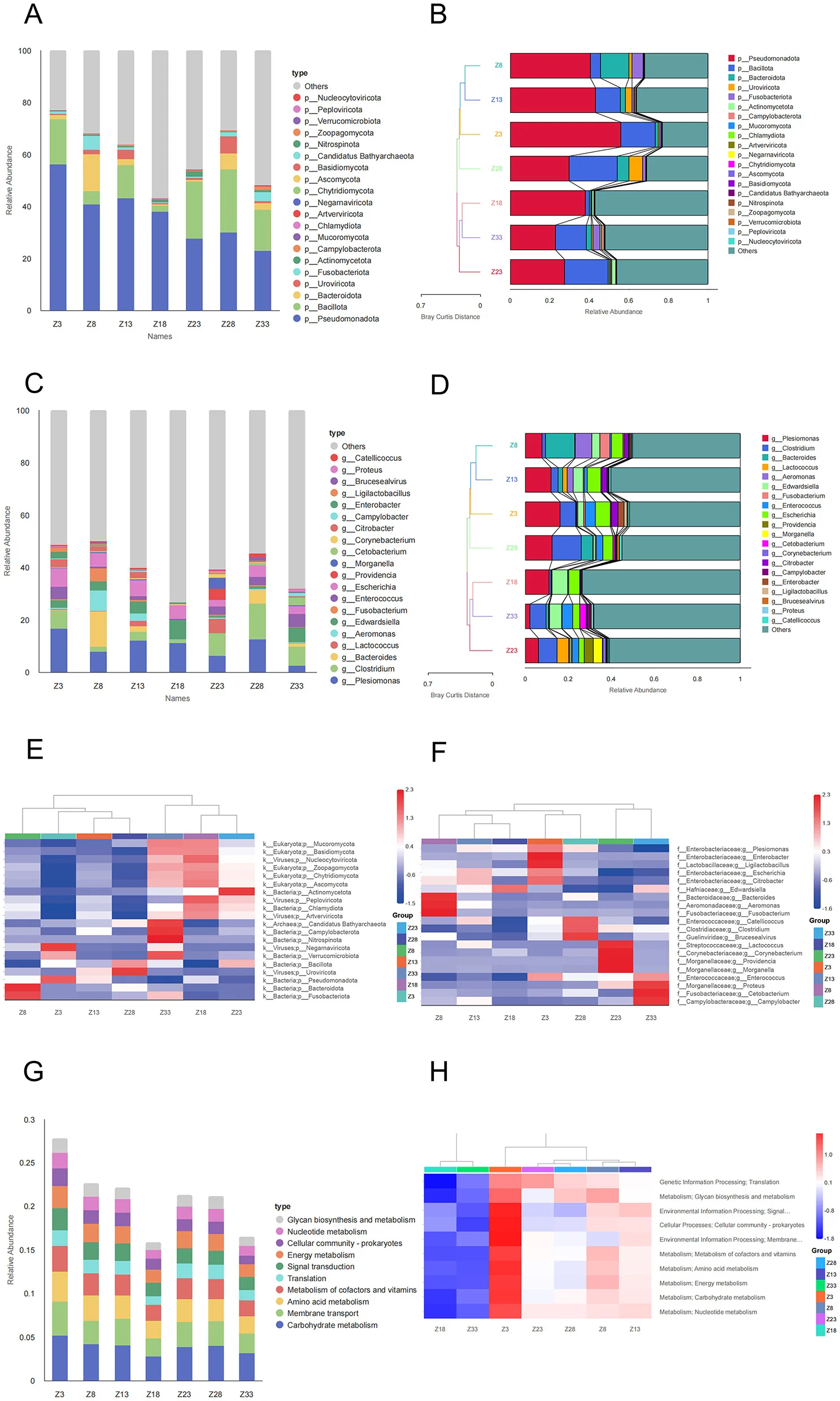
The proportions and differences of the bacterial communities in crested ibis. **(A)** Based on the species annotation results at the Phylum level, the top 20 species with the highest relative abundance for each group are selected, and the remaining species are set as “Others”. Bar charts of the relative abundance of species annotation results at different classification levels for each group are then plotted. **(B)** Clustertree of the top 20 species at the Phylum level. **(C)** Based on the species annotation results at the Genus level, the top 20 species with the highest relative abundance for each group are selected, and the remaining species are set as “Others”. Bar charts of the relative abundance of species annotation results at different classification levels for each group are then plotted. **(D)** Clustertree of the top 20 species at the Genus level. **(E)** Cluster heatmap of the top 20 species at the Phylum level. **(F)** Cluster heatmap of the top 20 species at the Genus level. **(G)** The proportions of the top 10 KEGG functional categories. **(H)** Clustertree of the top 10 KEGG functional categories.

At the genus level, relative abundance analysis indicated that the dominant bacteria in the crested ibis gut included *Plesiomonas, Clostridium*, and *Escherichia*. *Plesiomonas* exhibited high abundance in most groups, and *Clostridium* showed relatively high abundance in 23–33-day samples. The abundance of *Plesiomonas* was relatively high in the Z3 group, while the abundance of anaerobic bacteria such as Clostridium increased in some later-stage groups. Cluster analysis at the genus level further showed that samples of different ages formed different cluster branches in terms of microbial community structure. Among them, the difference between Z3 and other groups was more obvious, suggesting that the gut microbiota undergoes dynamic changes during the growth and development of the host (Figures 3C, D, Supplementary Table S5).

The microbial community clustering heatmap results further revealed the differences in microbial abundance among different samples. In the phylum-level clustering heatmap, *Pseudomonadota* and *Negarnaviricota* were more abundant in Z3 group, while the *Nitrospinota, Campylobacterota*, and *Candidatus Bathyarchaeota* was relatively high in the Z33 group (Figure 3E). In the genus level clustering heatmap, *Ligilactobacillus* and *Enterobacter* showed more enrichment in Z3 group, while *Cetobacterium, Proteus*, and *Campylobacter* showed high abundance in Z33 groups. Cluster analysis results showed obvious differences in microbial composition among samples of different ages, reflecting the continuous structural adjustments of the gut microbiota during development (Figure 3F).

In terms of functional analysis, annotation of gut microbial functions based on the KEGG database revealed that the overall functional composition of the gut microbiota. The relative abundance histogram showed that gut microbial functions in samples of different ages were mainly concentrated in pathways such as carbohydrate metabolism, amino acid metabolism, energy metabolism, membrane transport, nucleotide metabolism, cofactor and vitamin metabolism, translation, and cellular community-prokaryotes. Among these, carbohydrate metabolism and amino acid metabolism-related functions accounted for a relatively high proportion, indicating that gut microbes have a strong ability to break down complex carbohydrates and proteins, and these metabolic processes can provide the host with necessary energy and nutrients. Furthermore, the presence of energy metabolism pathways indicates that gut microbes can participate in energy conversion processes through multiple metabolic pathways, while membrane transport-related functions reflect the ability of microbial cells to take up nutrients and excrete metabolic products through multiple transport systems (Figure 3G).

Between the Z3 and the Z33 groups after hatching, the functional abundance of the intestinal microbiota of the crested ibises showed obvious difference. In the heatmap, the Z3 samples were at high abundance (dark red) in almost all the listed secondary functional pathways, while the Z33 samples were generally at low abundance (blue) (Figure 3H). The carbohydrate metabolism, energy metabolism and nucleotide metabolism in the Z3 stage were higher than those in the Z33 stage. This indicates that the early colonizing bacterial communities (usually facultative anaerobes such as Enterobacteriaceae, etc.) are using the yolk remnants or the initial artificial diet for explosive proliferation. The high abundance of amino acid metabolism and coenzyme and vitamin metabolism in Z3 suggests that the early microbiota played a crucial role in synthesizing essential nutrients for the host (such as B vitamins) and assisting in protein digestion. In contrast, by the 33rd day (Z33), as the intestinal environment became more stable and the diet structure changed, the relative abundance of these basic metabolic functions in the microbiota decreased, possibly shifting to a more specific metabolic pattern. The high abundance on the third day reflects the versatility of the pioneer colonizing microbiota. In the early stage of ecological niche vacancy, the colonizing microbiota often has an extremely active and comprehensive metabolic profile to ensure survival in the complex intestinal environment. As the crested ibis grows to the 33rd day, the intestinal microecosystem becomes increasingly mature. Although the abundance decreases on the heatmap, this does not indicate a loss of function; rather, it may imply a trend toward specialization. The mature microbiota operates through more efficient metabolic pathways, reducing the all-encompassing and energy-intensive functional expression seen in the early stage. The 3-day-old stage is a critical period for the crested ibis to transition from endogenous nutrition (yolk) to exogenous nutrition (artificial feeding), and at this time, the high metabolic activity of the microbiota is crucial for the survival of the host. By the 33rd day, the crested ibis has entered the growth mid-stage, with relatively well developed intestinal barriers, and the functional role of the microbiota shifts more from assisting development to maintaining immune balance and efficient feed conversion. According to the KEGG pathway abundance heatmap, there are extremely differences in function between the Z3 group and the Z33 group, showing almost completely opposite patterns. Metabolic pathways are the section with the most differences. The basal metabolism and secondary metabolism activities of the Z3 group far exceed those of the Z33 group. Specifically, they include: Energy metabolism, Carbohydrate metabolism, Amino acid metabolism, Nucleotide metabolism, Metabolism of cofactors and vitamins, as well as glycan biosynthesis and metabolism. This indicates that the Z3 group is undergoing intense processes of substance synthesis, energy decomposition and conversion, and is in a period of vigorous growth.

In terms of differential microbial community analysis, LEfSe analysis results showed that there were multiple significantly different microbial groups among different age groups. The LDA score bar chart used LDA score (log10) to represent the contribution of each differentially expressed microbial group to the differences between groups; a higher value indicated a stronger discriminative ability of the microbial group within the corresponding group. The results showed that significantly enriched dominant microbial groups existed at all age stages. The Z3 group was enriched with bacteria including Enterobacter, Citrobacter, Enterobacteriaceae, Clostridium, etc. The Z8 group was enriched with Aeromonas, Bacteroides, etc. The Z13 group was enriched with Uroviricota, Caudoviricetes_sp, etc. The Z18 group showed significant enrichment with Bifidobacterium and Bifidobacteriales. The Z23 group was enriched with Lactococcus, Corynebacter, etc. The Z28 group was enriched with Catellicoccus, Guelinviridae, etc. The Z33 group was enriched with Bacteroidaceae, Campylobacte_volucris, Moraxellales, Cetobacterium, etc. The enrichment of these differentially expressed bacterial groups at different age stages indicates that the gut microbiota undergoes continuous turnover as the host develops, with different microbial groups playing different ecological functions at different stages (Figure 4A).

**Figure 4:**
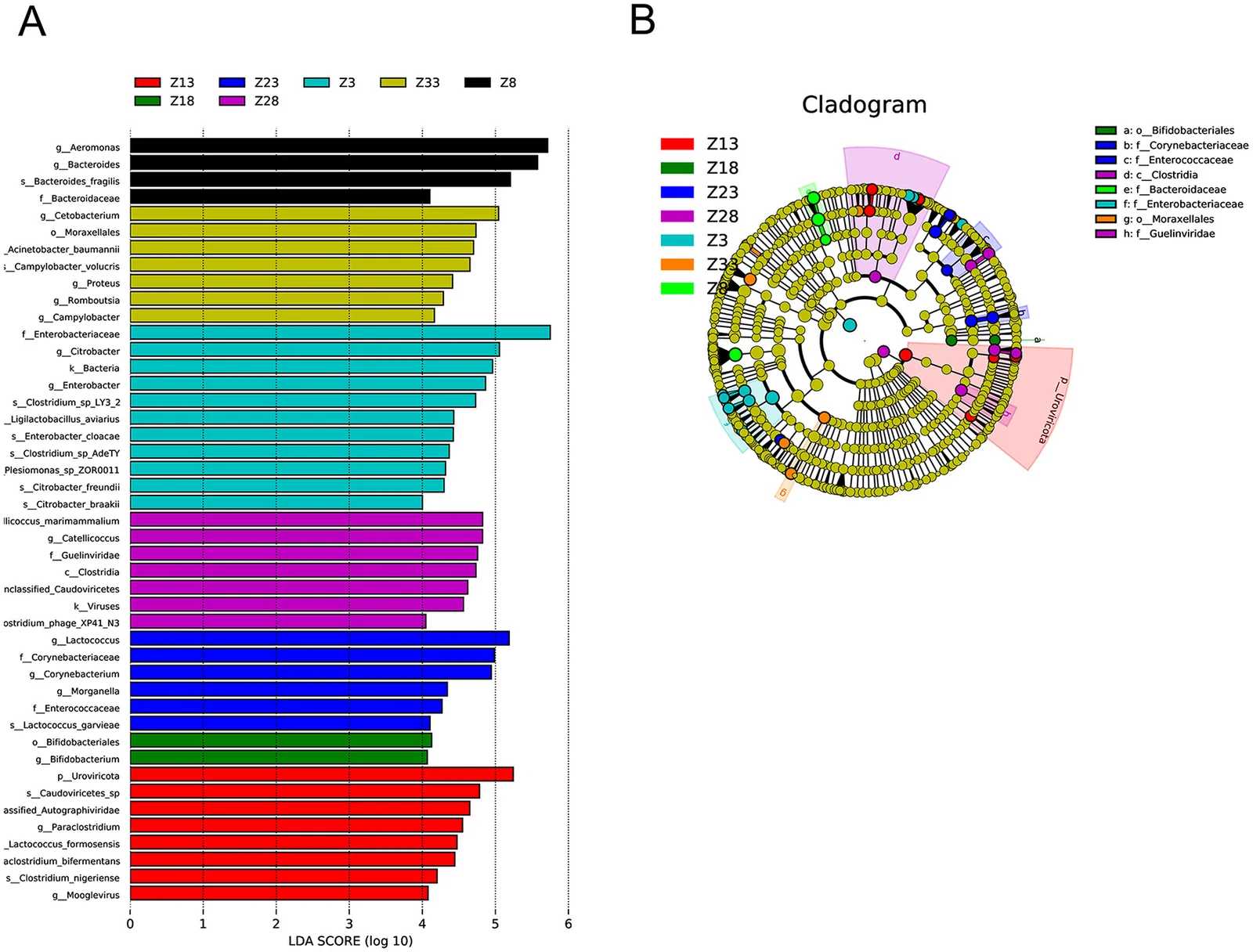
LEfSe analyses and evolutionary branching diagram of microorganisms. **(A)** LEfSe analyses of different groups. LDA (linear discriminant analysis) discriminant histogram. The LDA score obtained by LDA analysis indicates that the greater the LDA score, the greater the influence of species abundance on the difference effect. **(B)** Evolutionary branch diagram (unique phylogenetic distribution of species) and biomarkers with statistically significant differences between groups in the abundance comparison among different groups.

The Cladogram illustrates the distribution of differentially expressed gut microbiota at different taxonomic levels from a phylogenetic perspective. The clade, from the center outwards, represents different taxonomic levels from phylum, class, order, family to genus. Nodes of different colors indicate microbial groups significantly enriched in different age groups, while yellow nodes represent groups with no significant differences between groups. The clade shows that most differentially expressed gut microbiota are concentrated in phylogenetic branches such as Enterobacteriaceae, Enterococcaceae, Bacteroidaceae, Moraxellales, Bifidobacteriales, and Clostridia. Bifidobacteriales forms a distinct differential branch in group Z18, while Enterobacteriaceae is more prominent in earlier age groups. These results indicate significant differences in the phylogenetic structure of the gut microbiota at different age stages, and also reflect the complex ecological succession process that the gut microbiota undergoes during host development. Overall, differential microbiota analysis not only revealed the changing characteristics of microbiota composition among different age groups, but also provided important evidence for further understanding the gut microecological structure and potential physiological functions of crested ibises (Figure 4B).

### 3.4 Compositions and differential untargeted metabolites in the intestine of *Nipponia nippon*

In metabolomics analysis, the detected metabolites were first classified and statistically analyzed. The classification results under anion and cationic modes showed that the metabolites in the samples were mainly divided into lipids and lipid-like molecules, organic heterocyclic compounds, organic acids and derivatives, benzenoids, organic oxygen compounds, and phenolicpropanoids and polyketides. Among these, lipids and lipid-like molecules accounted for the highest proportion in both anion and cationic modes, followed by organic heterocyclic compounds and organic acids and derivatives. This result indicates that lipid metabolism-related substances and organic acids occupy a major position in the composition of intestinal metabolites of crested ibises, suggesting that intestinal metabolic activities are closely related to lipid metabolism, energy metabolism, and organic acid metabolism (Figures 5A, B).

**Figure 5:**
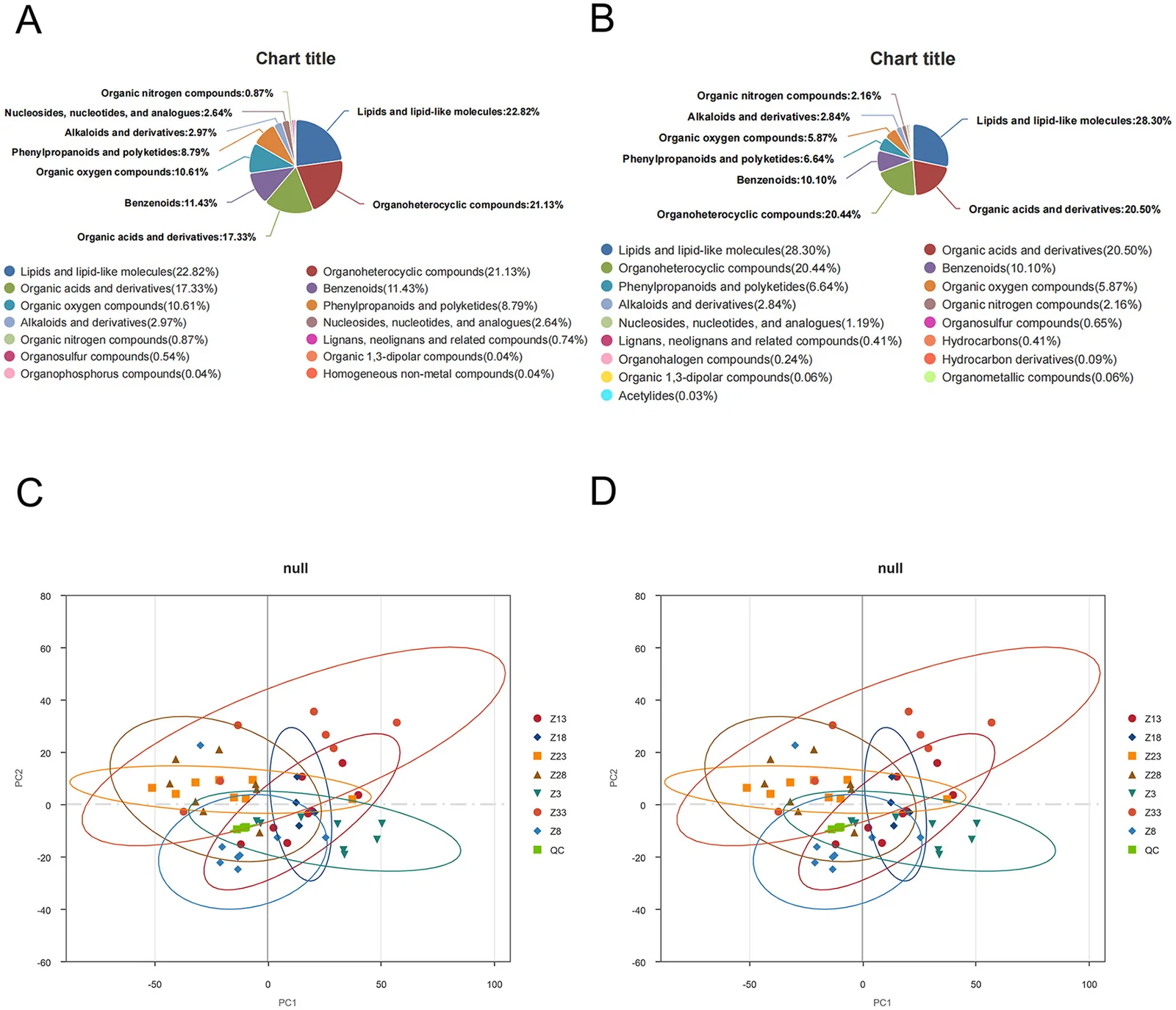
Metabolite classification of crested ibis fecal. **(A)** Based on the classify file database, all the metabolites identified in this project were annotated. The pie chart shows the anion mode classification and the proportion of metabolites contained in each classification. **(B)** The pie chart shows the cationic mode classification and the proportion of metabolites contained in each classification. **(C)** PCA analysis of the metabolites of anion mode. **(D)** PCA analysis of the metabolites of cationic mode.

PCA analysis further reflects the differences in overall metabolite composition among samples from different age groups. Anion-mode PCA analysis showed that all QC samples are tightly clustered in the plot, nearly overlapping and located near the origin of the coordinate axes. Although there is overall overlap, the distribution spans vary across different groups. For example, the ellipse for group Z33 (orange-red dots) not only covers a larger area but also extends distinctly toward the upper right corner of the plot; in contrast, earlier groups such as Z3 or Z8 show relatively more concentrated distributions. Cation-mode PCA analysis showed that Scatter points representing different groups (Z3 to Z33) and their corresponding 95% confidence intervals (ellipses in various colors) are highly overlapping on the two-dimensional plane. No group can be completely separated from the others. Meanwhile, QC samples clustered relatively tightly in the PCA plot, indicating good stability and repeatability of the metabolomics data and high reliability of the experimental results (Figures 5C, D).

Differential metabolite analysis used group Z3 as the control group and compared it with groups Z8, Z13, Z18, Z23, Z28, and Z33. The left side shows the differentially regulated metabolites detected in anion mode, and the right side shows the differentially regulated metabolites detected in cation mode. In the figure, red indicates up-regulated metabolites relative to group Z3, and blue indicates down-regulated metabolites. The horizontal axis is Log2FC, which represents the fold change in metabolite levels.

In the comparison between Z3 and Z8. In the cation mode, 27 metabolites were significantly upregulated, and 625 metabolites were significantly downregulated (Figure 6A, Supplementary Table S6). In the cation mode, 145 metabolites were significantly upregulated, and 215 metabolites were significantly downregulated (Figure 6B, Supplementary Table S7).

**Figure 6:**
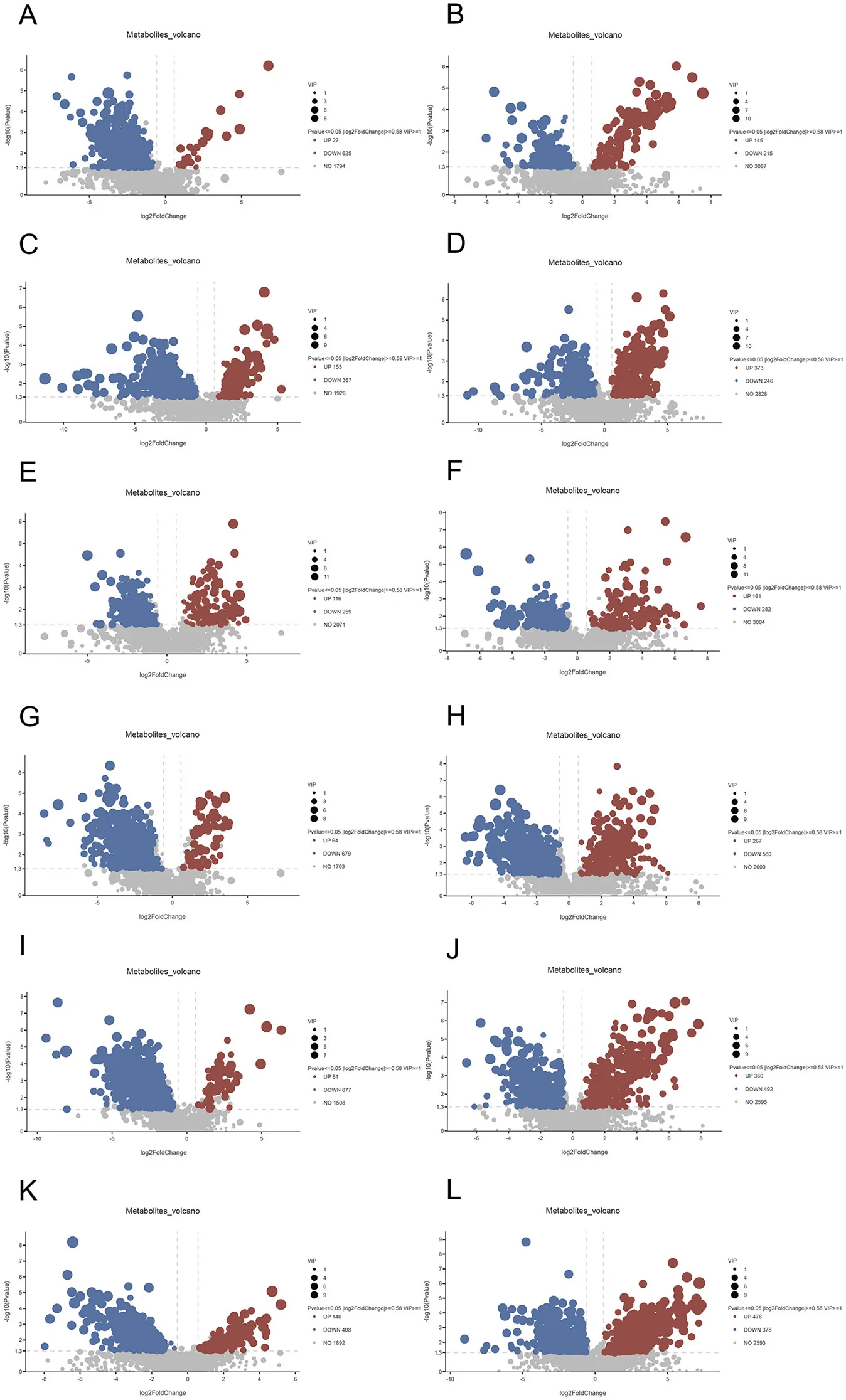
Volcano plot of differential metabolites between groups. **(A)** The differential metabolites between Z3 vs Z8 groups in the anion mode. The colors are used to distinguish the upregulation (red) and downregulation (blue) of the metabolites, The x-axis shows log2 (fold change): values farther from 0 indicate larger fold differences. The y-axis shows –log10 (*P*-value): higher values indicate smaller *P*-values and greater statistical significance. Dot size reflects VIP, with larger dots indicating greater contribution to group separation. **(B)** The differential metabolites between Z3 vs. Z8 groups in the cationic mode. **(C)** The differential metabolites between Z3 vs. Z13 groups in the anion mode. **(D)** The differential metabolites between Z3 vs. Z13 groups in the cationic mode. **(E)** The differential metabolites between Z3 vs. Z18 groups in the anion mode. **(F)** The differential metabolites between Z3 vs. Z18 groups in the cationic mode. **(G)** The differential metabolites between Z3 vs. Z23 groups in the anion mode; and **(H)** The differential metabolites between Z3 vs. Z23 groups in the cationic mode. **(I)** The differential metabolites between Z3 vs. Z28 groups in the anion mode. **(J)** The differential metabolites between Z3 vs. Z28 groups in the cationic mode. **(K)** The differential metabolites between Z3 vs. Z33 groups in the anion mode. **(L)** The differential metabolites between Z3 vs. Z33 groups in the cationic mode.

In the comparison between Z3 and Z13. the number of differentially regulated metabolites further increased. In the cation mode, 153 metabolites were significantly upregulated, and 367 metabolites were significantly downregulated (Figure 6C, Supplementary Table S8). In the cation mode, 373 metabolites were significantly upregulated, and 246 metabolites were significantly downregulated (Figure 6D, Supplementary Table S9). This result indicates that when the crested ibis reaches 13 days of age, its metabolic activity is significantly enhanced, with an increase in both the types and magnitude of metabolites involved.

In the comparison between Z3 and Z18. In the cation mode, 116 metabolites were significantly upregulated, and 259 metabolites were significantly downregulated (Figure 6E, Supplementary Table S10). In the cation mode, 161 metabolites were significantly upregulated, and 282 metabolites were significantly downregulated (Figure 6F, Supplementary Table S11). Overall, the Z18 stage showed more changes in lipid and organic acid-related metabolites, suggesting that lipid metabolism and energy metabolism processes become increasingly active with age.

In the comparison between Z3 and Z23. In the cation mode, 64 metabolites were significantly upregulated, and 679 metabolites were significantly downregulated (Figure 6G, Supplementary Table S12). In the cation mode, 267 metabolites were significantly upregulated, and 580 metabolites were significantly downregulated (Figure 6H, Supplementary Table S13). The significant changes in differentially expressed metabolites at this stage suggest that intestinal metabolic activity may undergo substantial adjustments at 23 days of age.

In the comparison between Z3 and Z28. In the cation mode, 61 metabolites were significantly upregulated, and 877 metabolites were significantly downregulated (Figure 6I, Supplementary Table S14). In the cation mode, 360 metabolites were significantly upregulated, and 492 metabolites were significantly downregulated (Figure 6J, Supplementary Table S15). Overall, the metabolite changes in the Z28 phase involved various types of organic acids, lipids, and alkaloids.

In the Z3 vs. Z33 comparison. In the cation mode, 146 metabolites were significantly upregulated, and 408 metabolites were significantly downregulated (Figure 6K, Supplementary Table S16). In the cation mode, 476 metabolites were significantly upregulated, and 378 metabolites were significantly downregulated (Figure 6L, Supplementary Table S17).

A small number of putative exogenous compounds, including synthetic pharmaceuticals and pesticides (Deflazacort, Sertraline, Mefloquine, Chloroxuron, and Molindone, et al.), were detected among the differential metabolites. These likely originated from feed, drinking water, or environmental exposure, and were not considered as endogenous developmental markers. They were excluded from subsequent pathway enrichment analysis and did not contribute to the reported pathway changes.

Overall, a large number of significantly differential metabolites were detected in all comparison groups, and the types and magnitudes of these differential metabolites showed an increasing trend with increasing age. The results of anion and cation detection were generally consistent, indicating significant differences in metabolic activity among crested ibises at different developmental stages. The differential metabolites mainly involved various metabolic types, including lipid metabolism, organic acid metabolism, amino acid metabolism, and secondary metabolites, suggesting that the metabolic level of crested ibises undergoes dynamic changes as they grow and develop, gradually forming relatively stable metabolic characteristics.

Pathway enrichment analysis was conducted for the groups, revealing the main pathways enriched in each age group relative to the Z3 group. The main enriched pathways in the Z8 group compared to the Z3 group were Biosynthesis of cofactors and Tyrosine metabolism (anion mode, Figure 7A, Supplementary Table S18) and metabolism pathways (cationic mode, Figure 7B, Supplementary Table S19), as identified through *P*-value analysis. The main enriched pathways in the Z13 group compared to the Z3 group were metabolic pathways and Bile secretion (anion mode, Figure 7C, Supplementary Table S20), metabolic pathways and steroid biosynthesis in cationic mode (Figure 7D, Supplementary Table S21), as identified through *P*-value analysis. The main enriched pathways in the Z13 group compared to the Z3 group were Biosynthesis of cofactors and ABC transporters (anion mode, Figure 7E, Supplementary Table S22); Biosynthesis of cofactors (cationic mode, Figure 7F, Supplementary Table S23), as identified through *P*-value analysis. The main enriched pathways in the Z23 group compared to the Z3 group were 2-Oxocarboxylic acid metabolism (anion mode, Figure 7G, Supplementary Table S24) and Biosynthesis of cofactors, Galactose metabolism, and bile secretion (cationic mode, Figure 7H, Supplementary Table S25), as identified through *P*-value analysis. The main enriched pathways in the Z28 group compared to the Z3 group were Biosynthesis of cofactors, Glyoxylate and dicarboxylate metabolism, and Cysteine and methionine metabolism (anion mode, Figure 7I, Supplementary Table S26); Nucleotide metabolism, Lysine biosynthesis, Steroid biosynthesis, and Neomycin, kanamycin, and gentamicin biosynthesis (cationic mode, Figure 7J, Supplementary Table S27), as identified through *P*-value analysis. The main enriched pathways in the Z33 group compared to the Z3 group were Nucleotide metabolism, Carbon metabolism, Biosynthesis of amino acids and ABC transporters (anion mode, Figure 7K, Supplementary Table S28); Metabolic pathways and Bile secretion (cationic mode, Figure 7L, Supplementary Table S29), as identified through *P*-value analysis.

**Figure 7:**
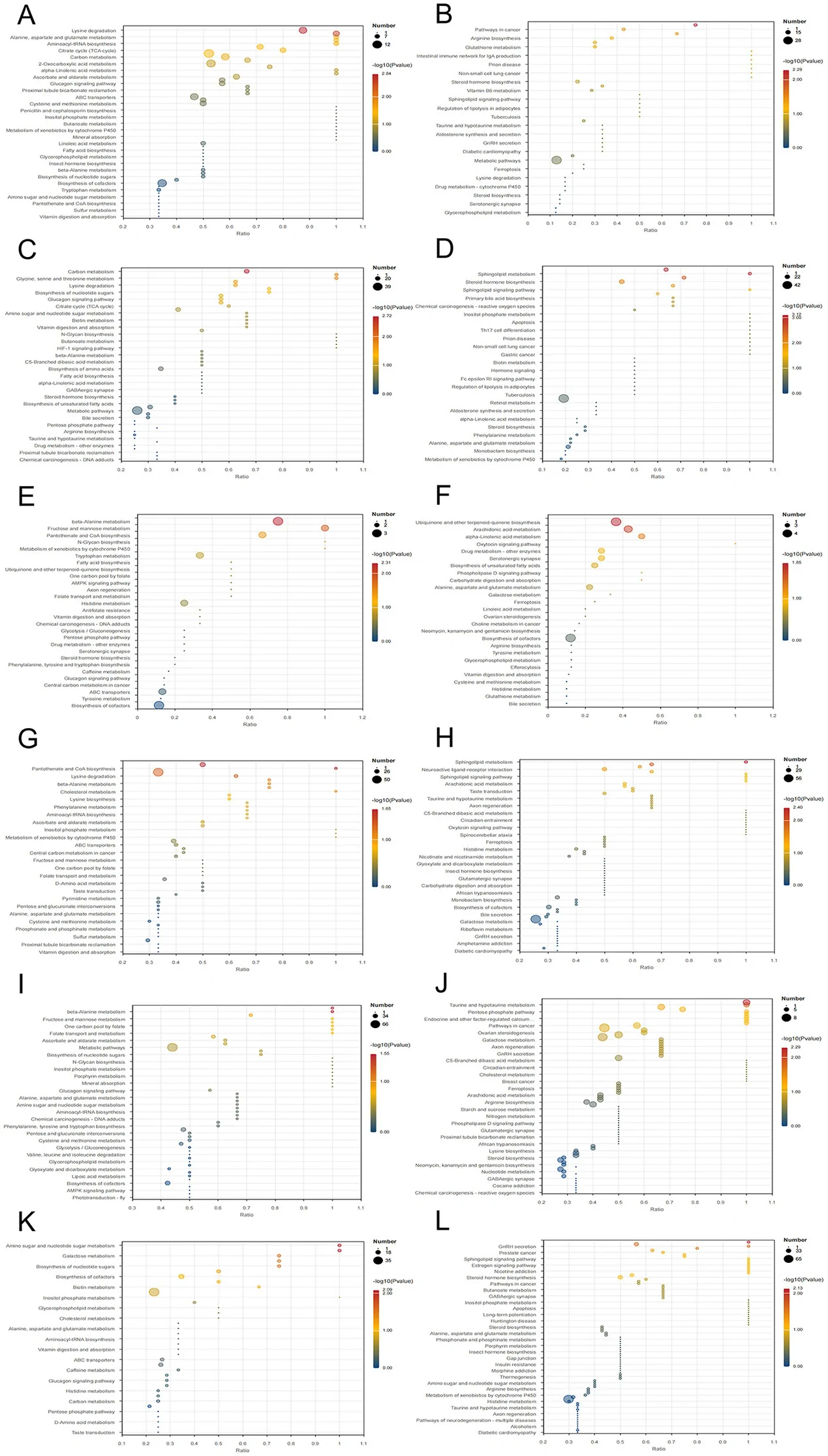
Top 20 KEGG enrichment analyses. **(A)** Top 20 KEGG enrichment analysis between Z3 vs. Z8 groups in the anion mode (*P*-value method). The horizontal coordinate is the enrichment significance *P*-value; generally, the size of the bubbles in the figure represents the amount of concentration of metabolic compound in this pathway. **(B)** Top 20 KEGG enrichment analysis between Z3 vs. Z8 groups in the cationic mode. **(C)** Top 20 KEGG enrichment analysis between between Z3 vs. Z13 groups in the anion mode (*P*-value method). **(D)** Top 20 KEGG enrichment analysis between Z3 vs. Z13 groups in the cationic mode. **(E)** Top 20 KEGG enrichment analysis between Z3 vs. Z18 groups in the anion mode (*P*-value method). **(F)** Top 20 KEGG enrichment analysis between Z3 vs. Z18 groups in the cationic mode. **(G)** Top 20 KEGG enrichment analysis between Z3 vs. Z23 groups in the anion mode (*P*-value method). **(H)** Top 20 KEGG enrichment analysis between Z3 vs. Z23 groups in the cationic mode. **(I)** Top 20 KEGG enrichment analysis between Z3 vs. Z28 groups in the anion mode (*P*-value method). **(J)** Top 20 KEGG enrichment analysis between Z3 vs. Z28 groups in the cationic mode. **(K)** Top 20 KEGG enrichment analysis between Z3 vs. Z33 groups in the anion mode (*P*-value method). **(L)** Top 20 KEGG enrichment analysis between Z3 vs. Z33 groups in the cationic mode.

### 3.5 Correlations between intestinal microbes and metabolites

A Pearson correlation analysis between samples was performed at the genus level. Compared to the Z3 group, the significant markers in the Z33 group were *Proteus, Klebsiella, Enterobacter, Corynebacterium*, and *Citrobacter*. In the anion mode, the metabolites Amoenin 6,7-Dihydroxy-5-methoxycoumarin 6-beta-D-glucopyranoside, Dracunculifoside J, Isoscrophularioside, Methyl mycophenolate, alpha-glutamyltyrosine, alpha-D-xylosyl-(1->6)-beta-D-glucosyl-(1->4)-beta-D-glucose, Lauryl sulfate, and m-xylene-4-sulfonic acid were positively correlated with *Corynebacterium*, however these metabolites were negatively correlated with *Klebsiella, Enterobacter*, and *Citrobacter;* the metabolite Isariotin C was negatively correlated with *Corynebacterium*, but positively correlated with *Klebsiella, Enterobacter*, and *Citrobacter* (Figure 8A), the significance of the correlation can be found in Supplementary Table S30. In the cationic mode, the metabolites 4-Mercapto-5-methyl-3(2H)-thiophenone, Triethylamine were positively correlated with *Corynebacterium*, however the metabolites were negatively correlated with *Klebsiella, Enterobacter*, and *Citrobacter;* the metabolites 3′-hydroxy-alpha-tocopherol, 2-Amino-2-norbornanecarboxylic acid, Deflazacort, Dendroside A, Fatsicarpain F, 2-(3,4-dimethoxyphenyl)-1-{3-[(3,5-dimethylpiperidin-1-yl)methyl]-2,4-dihydroxyphenyl} ethan-1-one, Hexadecanoylpyrrolidine, Urechitol B were negatively correlated with *Corynebacterium*, but positively correlated with *Klebsiella, Enterobacter*, and *Citrobacter* (Figure 8B), the significance of the correlation can be found in Supplementary Table S31.

**Figure 8:**
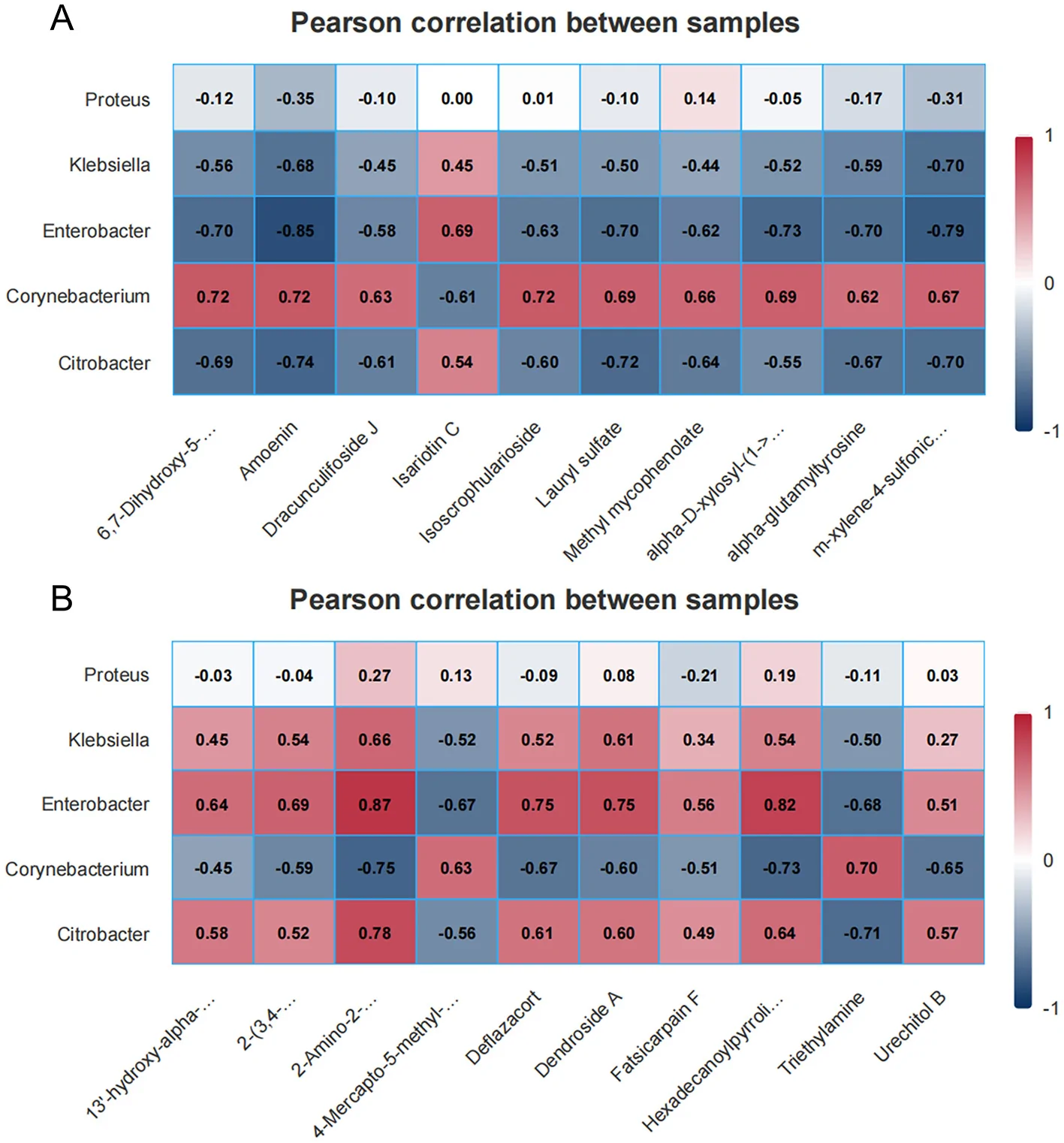
Differential microbial and differential metabolite association analysis. **(A)** The microorganism and metabolite association analysis in the anion mode. Based on the Pearson correlation coefficient, a correlation analysis was conducted between the significantly different bacterial communities identified by microbial analysis and the significantly different metabolites obtained from metabolomics analysis. When the changes in metabolites are in line with those of a certain bacterial community, they show a positive correlation; conversely, if the trends are opposite, they exhibit a negative correlation. **(B)** The microorganism and metabolite association analysis in the cationic mode.

## 4 Discussion

For the *Nipponia nippon*, as a globally rare and endangered bird species, the early growth and development of its young birds directly affect the breeding success rate and conservation effectiveness of the population. The early stage after hatching is not only a critical developmental window for the rapid maturation of the bird's immune system and digestive function, but also an important stage for the colonization, succession, and stability of its intestinal microecosystem. However, there is currently a lack of systematic understanding of how the intestinal microbial community of crested ibises interacts dynamically with the host's metabolites during this specific developmental period. This study comprehensively utilized metagenomics and metabolomics techniques to deeply analyze the phased succession characteristics of the fecal microbiota of naturally hatched reared crested ibises from 3 to 33 days after hatching. Our research not only confirmed that as the age of the chicks increased, the core bacterial community structure and metabolic pathways in their intestines underwent significant and regular remodeling, but also revealed the potential associations between specific microbial groups and key metabolites. These findings fill the gap in the research on the intestinal microecology of crested ibises during their early development stage, providing a solid theoretical basis for in-depth understanding of their early nutritional and metabolic mechanisms, optimizing artificial breeding and feeding strategies, and improving the early survival rate of chicks.

The intestinal microecology shows extremely high dynamic instability during the early stage of the juvenile bird's development. This characteristic was clearly confirmed in the α-diversity results of this study. The results showed that from the 3rd day after hatching (Z3) to the 13th day (Z13), the abundance (ACE and Chao1 index) and diversity (Shannon index) of the fecal microbiota of the crested ibis chicks exhibited an upward trend, and reached a peak at around Z13. This early increase in diversity may be closely related to the rapid increase in the chicks' food intake, the rapid development of the digestive tract morphology, and the expansion of the contact surface with the external environment (such as nest materials and artificial feeding tools). It is worth noting that from Z13 to Z33, the diversity index did not continue to increase unidirectionally but fluctuated and gradually approached a plateau. This phenomenon conforms to the classic theory of microbial community succession: as the large-scale colonization of “pioneer species” occurs, the intestinal ecological niche gradually becomes saturated, competition for nutrients and spatial exclusion intensify, resulting in the elimination of some passing bacteria. This indicates that the intestinal microecosystem of the crested ibises is transitioning from the initial “blind expansion” toward a more mature and orderly self-regulating state.

In addition to the changes in community richness, age is the decisive factor that drives the macroscopic structure remodeling of the intestinal microbiota of juvenile crested ibises. The principal coordinate analysis (PCoA) at the phylum and genus levels visually reveals this evolutionary trajectory over time. Particularly in the beta diversity analysis at the genus level, the samples from the extremely early stage of development (Z3) are distinct from those from the middle and later stages on the coordinate axes, presenting a relatively independent separation. This indicates that the intestinal microbial composition at the early post-hatching stage has a high degree of uniqueness, and the intestines at this time are mostly in a facultative anaerobic environment. As the age increases (from Z13 to Z33), the sample points gradually shift and overlap in space, indicating a fundamental restructuring of the microbiota structure. This progressive evolution from the “pioneer community” in the early stage to the “mature community” in the later stage reflects the strong selection pressure exerted by the intestinal microenvironment (such as the formation of a strict anaerobic environment, changes in pH value) on microorganisms.

Metagenomic genetic diversity in the gut mainly determines what functions the microbiota can perform and how resilient the ecosystem is to disturbance, which in turn affects host nutrition, immunity, and disease risk (Wang et al., 2015). Gut microflora plays a crucial role in the health and production performance of animals. We analyzed the composition and taxa from 3 to 33 days in the feces of *Nipponia nippon*. At the phylum level, Pseudomonadota and Bacillota were the dominant bacteria. Pseudomonadota is a large and diverse bacterial phylum whose members perform many different ecological and biological functions rather than a single function. Many species within Pseudomonadota participate in global carbon, nitrogen, and sulfur cycles, for example by oxidizing ammonia, reducing nitrate, or degrading organic compounds in soil and water (Chuai et al., 2026). Bacillota also does not have one single function; its members perform many different roles in ecosystems and in hosts such as plants and animals. Many members of Bacillota (such as *Bacillus, Clostridium*, and *Paenibacillus*) break down complex plant polymers like cellulose, hemicellulose, and pectin, helping to decompose organic matter and recycle carbon in soils and aquatic environments (Mandic-Mulec et al., 2015). Bacillota includes aerobes, facultative anaerobes, and strict anaerobes, with fermentative, chemoorganotrophic, and some photoheterotrophic lifestyles, allowing them to inhabit many niches from soils to sediments to animal guts (Endress et al., 2024).

From a functional perspective, different dominant bacterial phyla play important roles in the gut ecosystem. Pseudomonadota, with its strong environmental adaptability and metabolic diversity, participates in the degradation of various organic substances and energy metabolism processes, playing a crucial role in early microbial colonization and environmental adaptation in the host gut. Bacillota contains a large number of bacteria capable of carbohydrate fermentation, whose metabolic processes produce metabolites such as short-chain fatty acids. These substances not only provide energy sources for the host but also play an important role in maintaining gut microbiota balance and regulating host immunity. Bacteroidota is known for its strong polysaccharide degradation capacity, capable of breaking down complex carbohydrates and promoting nutrient conversion and absorption, which is significant for host nutrient utilization and energy acquisition. Therefore, these dominant bacterial phyla synergistically participate in the decomposition, conversion, and energy metabolism of nutrients in the gut, playing a vital role in maintaining the stability of the crested ibis gut microbiota and the health of the host.

At the genus level, *Plesiomonas, Clostridium, Bacteroides, Edwardsiella*, and *Escherichia* were the dominant bacteria. The abundances of *Enterobacter* and *Ligilactobacillus* were decreased in the Z33 group compared to Z3 group, however, the abundances of *Proteus, Cetobacterium*, and *Campylobacter* were increased in the Z33 group compared to Z3 group.

*Enterobacter* is a genus of Gram-negative, facultatively anaerobic bacteria belonging to the family Enterobacteriaceae. In the gastrointestinal tract, it typically exists as a commensal organism in low abundance but functions as a highly adaptable opportunistic pathogen depending on the host's physiological state and the surrounding microbial ecology. Enterobacter is usually an opportunistic, facultative anaerobic bacterium that helps consume oxygen, which supports the low-oxygen environment needed by many normal gut microbes. Some strains may also help with nutrient processing and vitamin K-related metabolism, but its role is generally minor compared with the broader gut microbiota (Moreira de Gouveia et al., 2024). At low levels, Enterobacter-related bacteria can be part of a healthy ecosystem, but when they overgrow, they are often linked with dysbiosis and inflammation. In inflamed guts, they can gain an advantage because they tolerate oxygen and nitrate better than many beneficial anaerobes, and they may contribute to inflammatory signaling and barrier disruption. In a healthy gut, its oxygen-consuming activity can support other microbes; in disease states, especially inflammatory bowel conditions, excess Enterobacteriaceae are associated with worse inflammation and microbial imbalance (Baldelli et al., 2021).

*Ligilactobacillus* in the gut is generally considered a beneficial lactic-acid bacterium that helps support gut balance, barrier function, and immune regulation (Yang et al., 2024). A common example is Ligilactobacillus salivarius, which has been studied as a probiotic and has shown anti-inflammatory and barrier-protective effects in experimental models. Unlike opportunistic bacteria that can become problematic when they overgrow, Ligilactobacillus species are usually discussed as supportive members of the gut ecosystem (Yang et al., 2024; Carbonne et al., 2023; Yao et al., 2021).

*Proteus* in the gut is usually viewed as an opportunistic bacterium rather than a helpful core resident. It can persist in the intestine using motility, adhesion, and urease, and it may contribute to dysbiosis when it overgrows. Proteus can break down urea with urease, which produces ammonia and can alter the local gut environment, and evade host defenses with proteases, hemolysins, and other virulence factors. The bacteria often associates with gastrointestinal problems such as diarrhea, gastroenteritis, and inflammatory bowel disease in some studies (Hamilton et al., 2018). Unlike probiotic bacteria, Proteus is generally considered a pathobiont: it may be present without causing harm, but higher levels can be a sign of imbalance or inflammation (Drzewiecka, 2016).

*Cetobacterium* is generally considered a beneficial gut bacterium, especially in fish and other animals where it is common. Its main functions include producing acetate, helping with carbohydrate metabolism, supporting gut barrier health, and sometimes contributing to vitamin B12 production. The bacteria can protect against pathogens by strengthening gut defenses, improve digestion and nutrient use, particularly in fish diets (Qi et al., 2023; Wang et al., 2021; Ramírez et al., 2018).

*Campylobacter* in the gut is primarily a foodborne pathogen rather than a beneficial commensal. It is a leading bacterial cause of gastroenteritis worldwide, often producing watery or bloody diarrhea, abdominal pain, and nausea. It can cause inflammation and barrier disruption in the intestine, leading to enteritis and colitis (Rouhani et al., 2020). Campylobacter is generally considered a harmful enteropathogen in the gut, not a normal beneficial bacterium. Its presence usually signals infection or dysbiosis (Jalanka et al., 2023).

Through a correlation analysis of the metagenomic and metabolomic data, metabolites related to divergent microbiota were identified. In the anion mode, the five types of differential bacterial communities have formed two completely distinct alliances in terms of metabolic regulation. The positive correlation-driven type is Corynebacterium. This genus shows a significant positive correlation with the vast majority of metabolites, with the correlation coefficients ranging from 0.62 to 0.72. This indicates that under the current experimental conditions, an increase in the abundance of Corynebacterium may have promoted the accumulation of these metabolites, or these metabolites provide a crucial growth substrate for this bacterium.

The negative correlation dominant type is Enterobacteriaceae members (Enterobacter, Citrobacter, Klebsiella). These three bacterial genera exhibit a high degree of consistency, showing a strong negative correlation with most metabolites. Particularly, the Enterobacter genus (Enterobacter) has a negative correlation coefficient of −0.85 with Amoenin. This consistency suggests that these genera have similarities in their metabolic niches, possibly participating together in the degradation process of these metabolites, or these metabolites may have inhibitory effects on this type of bacterial community.

Isariotin C is the only metabolite in the graph whose correlation direction has reversed. It has a significantly negative correlation (-0.61) with Corynebacterium. It has positive correlations with Enterobacter (0.69), Citrobacter (0.54), and Klebsiella (0.45). Isariotin C is generally regarded as a secondary metabolite of fungi (Zhang et al., 2018). This reversal of correlation suggests a delicate antagonistic relationship in the intestinal microecology. For instance, the prosperity of bacterial groups such as Enterobacter may be accompanied by an increase in Isariotin C, and this environment may be unfavorable for the survival of Corynebacterium; or Isariotin C itself is a by-product of the metabolic activities of these Gram-negative bacteria (Enterobacteriaceae) (Yahagi et al., 2020).

The metabolites involved include a variety of complex chemical structures: sugars and glycosides along with plant chemical components, such as Dracunculifoside J, Isoscrophularioside, etc. These components usually come from the diet or feed. Corynebacterium has a positive correlation with these components, which may indicate that this bacterial community is involved in the preliminary processing of these complex organic substances. Amino acid derivatives, such as alpha-glutamyltyrosine, its negative correlation with Enterobacter and others (−0.73) may reflect the high consumption of proteins/amino acids by the intestinal microbiota. When the Enterobacteriaceae bacteria proliferate excessively, these nutritional metabolites are rapidly utilized, resulting in a decrease in their content.

There is a completely opposite metabolic association pattern between Enterobacter and Corynebacterium, which strongly proves that they are in different metabolic ecological niches in the intestinal tract. The strong negative correlation (−0.85) between Enterobacter and Amoenin can serve as an excellent combined biomarker for assessing the health status of the intestinal environment.

In the cationic mode, the combined analysis heatmap of metagenomics and metabolomics presents a completely different metabolic interaction pattern. The ecological niche competition relationship between the Enterobacter, Citrobacter, and Klebsiella genera and the Corynebacterium genus has been confirmed once again, but the direction has completely reversed.

Triethylamine and 4-Mercapto-5-methyl-3(2H)-thiophenone were highly dependent on Corynebacterium. These are the only two substances that are positively driven by Corynebacterium (0.70 and 0.63) and are strongly inhibited by the Enterobacteriaceae family. Triethylamine (TEA) is extremely crucial in intestinal microbiology. It is usually an intermediate product of the metabolism of nutrients such as choline and L-carnitine by the intestinal microbiota (Sugiyama et al., 2022; Zhai et al., 2023). There is a strong positive correlation between Corynebacterium and triethylamine, suggesting that this genus may play a central role in the host's choline metabolic network. If the abundance of this genus increases on the 33rd day, it indicates that the intestinal microecology has begun to possess the ability to handle more complex nutritional metabolism. 4-Mercapto-5-methyl-3(2H)-thiophenone is an extremely important sulfur-containing volatile heterocyclic compound (referred to as the core component of “meat flavor” in food chemistry). In organisms, its precursor is usually a sulfur-containing amino acid (such as methionine, cysteine) or thiamine (vitamin B1). Its appearance represents the metabolism of sulfur sources (sulfur-containing amino acids).

The simultaneous increase of these two indicates that Corynebacterium may play a central hub role in the deep fermentation (including deamination and deamination reactions) of proteins and amino acids in the lower part of the host's intestine. In addition, given that 4-mercaptomethyl-5-methyl-3(2H)-thiophenone often appears as a by-product of the Maillard reaction in heat-treated foods, the strong positive correlation with Corynebacterium also suggests that this bacterial group may have a special ecological adaptability to the digestion and metabolism of specific processed diets during the artificial rearing period. In contrast, the Enterobacteriaceae family (such as Enterobacter) has a strong negative correlation with these two metabolites, further confirming that there is a significant ecological niche exclusion and functional differentiation in the digestive system when dealing with these complex nitrogen/sulfur substrates.

## 5 Conclusion

This study integrates metagenomic and metabolomic analyses to characterize gut microbiota dynamics in naturally hatched crested ibis' chicks from 3 to 33 days post-hatching. Early phase (Z3) is dominated by facultative anaerobes (Enterobacter, Citrobacter) with high carbohydrate, amino acid, and energy metabolism. Diversity peaks at Z13–Z18, then stabilizes by Z28–Z33 with increased Cetobacterium and Campylobacter. Functional profiles are opposite between Z3 and Z33, reflecting a shift from versatile pioneer to specialized mature community. Correlation analysis reveals Corynebacterium positively associated with most metabolites, while Enterobacter, Citrobacter, and Klebsiella show negative correlations. Conservation implications include a 3–13-day probiotic intervention window, nutrition aligned with microbial capacity, and pathogen monitoring by day 33. This baseline enables evidence-based captive breeding improvements for this endangered species.

## Data Availability

The metagenomic sequencing reads analyzed in this study are available in the NCBI Sequence Read Archive (https://www.ncbi.nlm.nih.gov/) under BioProject accession number PRJNA1406017. The metabolomic raw data are available in the CNCB Genome Sequence Archive (https://ngdc.cncb.ac.cn/gsa/) under accession number OMIX014467.
