# Supplementary material for: Metagenomic and metabolomic analysis of the early gut microbiota development in Nipponia nippon
Source: Front Microbiol. 2026 Oct 8;17:1909232. doi: 10.3389/fmicb.2026.1909232 (PMC13687395; doi:10.3389/fmicb.2026.1909232)
Supplement: Supplementary file 1 [file Data_Sheet_1.zip › Table S1 Feed formula of Crested Ibis chick.docx]

Table S1 Feed formula of Crested Ibis chick

| Age (days) | Feed Number | Loach | Flour weevil | Egg yolk | Apple | Milk | Water |
| --- | --- | --- | --- | --- | --- | --- | --- |
|  |  | % | % | % | % | % | % |
| 1～6 | 1 | 50 | 6 | 6 | 5 | 7 | 26 |
| 7～12 | 2 | 68 | 10 | 4 | 4 | 4 | 10 |
| 13～20 | 3 | 75 | 11 | 3 | 3 | 3 | 5 |
| 21～40 | 44 | 82 | 10 | 2 | 2 | 2 | 2 |
